# SP-LL-37, human antimicrobial peptide, enhances disease resistance in transgenic rice

**DOI:** 10.1371/journal.pone.0172936

**Published:** 2017-03-10

**Authors:** In Hye Lee, Yu-Jin Jung, Yong Gu Cho, Ill Sup Nou, Md. Amdadul Huq, Franz Marielle Nogoy, Kwon-Kyoo Kang

**Affiliations:** 1 Department of Horticultural Life Science, Hankyong National University, Ansung, Korea; 2 Institute of Genetic Engineering, Hankyong National University, Ansung, Korea; 3 Department of Crop Science, Chungbuk National University, Cheongju, Korea; 4 Department of Horticulture, Sunchon National University, Suncheon, Korea; Fujian Agriculture and Forestry University, CHINA

## Abstract

Human LL-37 is a multifunctional antimicrobial peptide of cathelicidin family. It has been shown in recent studies that it can serve as a host’s defense against influenza A virus. We now demonstrate in this study how signal peptide LL-37 (SP-LL-37) can be used in rice resistance against bacterial leaf blight and blast. We synthesized LL-37 peptide and subcloned in a recombinant pPZP vector with pGD1 as promoter. SP-LL-37 was introduced into rice plants by *Agrobacterium* mediated transformation. Stable expression of SP-LL-37 in transgenic rice plants was confirmed by RT-PCR and ELISA analyses. Subcellular localization of SP-LL-37-GFP fusion protein showed evidently in intercellular space. Our data on testing for resistance to bacterial leaf blight and blast revealed that the transgenic lines are highly resistant compared to its wildtype. Our results suggest that LL-37 can be further explored to improve wide-spectrum resistance to biotic stress in rice.

## Introduction

As rice is the main food source for more than half of the world's population, fluctuation in production can indirectly make a significant impact on the world economy. Bacterial leaf blight and blast are the most important diseases affecting rice production. They are difficult to control because the speed of diffusion is very fast. The usual way to control such type of pathogens is to spray pesticides. However, continuous and extensive use of such pesticides causes long-term exposure of the environment to contamination. In addition, many microorganisms can acquire resistance to chemical pesticides [1]. The most effective and environment friendly way to control bacterial leaf blight and blast diseases is deployment of resistant cultivars. Classical plant breeding for resistant varieties requires complex system development like identification of source for donor genes, time and cost of breeding, stability of resistance and multiple location tests. Biotechnology can be used to complement the shortcomings of traditional breeding, and a number of success stories have already been reported.

Antimicrobial peptides (AMPs) are components of the defense system of animals and plants against pathogens and are generally shorter than 50 amino acid residues per peptide. AMPs are controlled by a single gene, and can destroy microorganisms, including bacteria, fungi, mycoplasma, and viruses, with minimum energy consumption during the initial infection. Because of this huge advantage, scientists used AMPs as one of the important sources for breeding resistant varieties [2]. The current plant pathogens have already evolved to have high resistance to the endogenous AMPs of plants. However, the microbial peptides of animal origin are less toxic than other types of peptides and they can inhibit pathogens faster and with greater efficiency.

Cecropins were isolated from the silkworm moth *Hyalophora cecropia* Linnaeus in 1980 and they were considered as AMPs derived from animals [3]. Rice transformed with codon-optimized cecropin A were shown to have increased resistance to blast disease [4]. Expression of foreign AMPs in the transformant played a catalytic role in the development of resistant varieties. Interestingly, however, transgenic potato and tobacco containing the cecropin B gene displayed sensitivity to soft rot caused by *Erwinia carotovora* and *Pseudomonas syringae* pv. tabaci [5–7]. Later, cecropin B peptides were found to be susceptible to degradation by peptidases in plants. The short persistence of cecropin B was reported as a result of initial intracellular endopeptidase or protease activity [8].

Aiming for production of a mature peptide using signal sequences of different origin has achieved a relatively high success rate [2, 9]. CECMEL 11 expression in rice was carried out through ER signal peptide, causing accumulation in the endoplasmic reticulum, to obtain partial resistance. However, the results observed under the microscope revealed that the organelles were exposed to heavy metal stress [10]. Use of a signal peptide for intercellular secretion is an optimal strategy that can avoid degradation by the plant proteases without adversely affecting the organelles and materials. The PR-1a signal peptide gene of tobacco was fused to sarcotoxin 1A-GUS fusion protein. Plants into which PR-1a was introduced displayed blocking of the protease degradation, and mature protein was secreted into the intercellular space [11].

LL-37 is the only member of the cathelicidin family of antimicrobial peptides isolated from humans. It is an amphipathic, 37-residue-long ɑ-helical peptide which has a broad spectrum of antibacterial activity [12]. The introduction of LL-37 into Chinese cabbage showed increased resistance to soft rot disease [13]. In this study, we report the stable transgenic expression of LL-37 peptide in Dongjinbyeo. In order to avoid degradation by the plant proteases, the fusion of vicilin at the N-terminal of LL-37 was carried out to allow secretion into the intercellular space. The pGD1 (Phosphogluconate dehydrogenase) promoter from rice was used to induce stable expression of SP-LL-37 in transgenic rice [14]. pGD1::SP-LL-37 intergenic lines were selected through FST (Flanking Sequence Tag) analysis, as differences in phenotype compared with the wildtype were not observed. The expression of SP-LL-37 peptide in rice plants significantly inhibited the growth of *Xanthomonas oryzae* in leaves that cause leaf blight disease. Transgenic plants also showed high resistance against blast disease caused by *Magnaporthe oryzae*. The SP-LL-37 transgenic rice will be a valuable breeding line for resistant variety development.

## Materials and methods

### Plant materials and growth conditions

Rice cultivar *Oryza sativa* L. var. Japonica cv. Dongjinbyeo (seeds obtained from the National Agrobiodiversity Center, RDA, Suwon, Korea) was used in plant transformation. There were 472 T_0_ plants used for PCR analysis to confirm the transformation.

The wild type Dongjinbyeo and transgenic lines were grown in paddy field condition. At maturity, agronomic traits were evaluated for chlorophyll content, days to flowering, plant height, culm length (CL), panicle length (PL), panicles per plant (PPP), spikelets per panicle (SPP), percent ripened grain (PRG), and 1000-grain weight (TGW). Evaluation methods were similar to that described in Cho et al. (2007) [15].

### *Agrobacterium* mediated transformation

**Fig 1:**
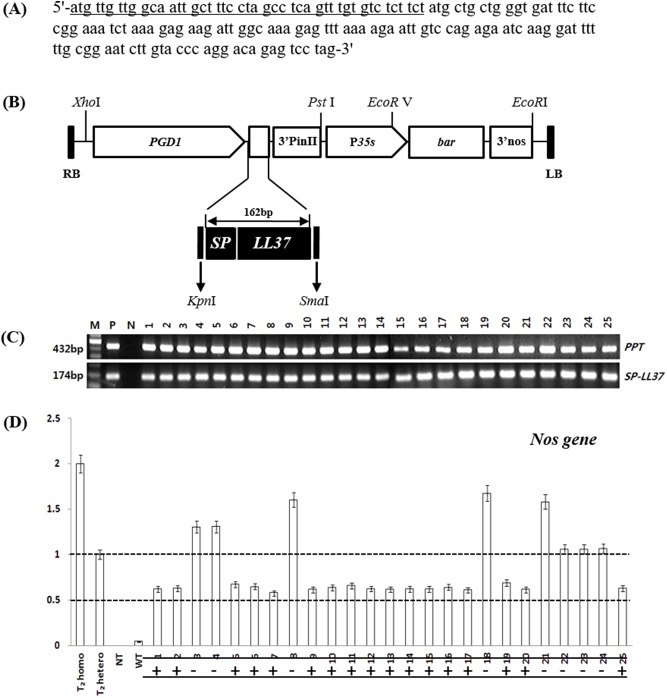
Sequence and the expression construct of SP-LL-37. (A) Nucleotide sequence of SP-LL-37 containing the coding region and stop codon as well as the signal peptide (underlined). (B) Schematic diagram of the expression construct pSP1::SP-LL-37. Detection of Nos gene copy numbers in the T_0_ generation of pPZP::SP-LL-37 plants. (C) PCR analysis of SP-LL-37 transgenic rice. Lanes: M; molecular marker, P; pPZP::SP-LL-37 vector, NT; non-transformed (NT) control, 1~25; independent T_0_ transgenic lines. (D) TaqMan PCR analysis for copy number assays using TaqMan probe for single copy selection in T_0_ transgenic Rice. +; single copy, -; multi copy, T_2_-homozygous and T_2_-heterozygous; single copy control, NT; negative control, WT; wild type control.

The recombinant binary pPZP vector was constructed by inserting SP-LL-37 synthetic gene in the *Kpn*I and *Sma*I enzyme sites. The whole sequence of LL-37 is shown in Fig 1A. SP-LL-37 was controlled by promoter pGD1 derived from Nakdongbyeo of japonica type, while the bar gene and nos terminator were used as selectable markers (Fig 1B). The construct was transformed into rice by *Agrobacterium* (*A*. *tumefaciens* strain EHA105) mediated transformation. Transgenic plants were grown in 1/2 MS (Murashige-Skoog) plates supplemented with 6 mg/L PPT to screen for PPT-resistant lines before cultivation in soil. Introduction of the gene in transgenic plants was confirmed by PCR using the primers 5’-ATGTTGTTGGCAATTGCTTTCC-3’ (Forward) and 5’- CTAGGACTCTGTCCTGGGTACAAG-3’ (Reverse) for the SP-LL-37 gene and all other primer sequences used for the subsequent experiments are listed in S1 Table.

### TaqMan real-time quantitative PCR (qPCR)

TaqMan real-time quantitative PCR was performed with a LightCycler® 480 machine and the LightCycler® 480 Probes Master kit (Roche). Reactions have a final volume of 10 μL composing of 1× LightCycler 480 Probes Master, 0.5 μM forward primer, 0.5 μM reverse primer, 0.2 μM TaqMan probes and 2.5 μL of the DNA template. Amplification conditions were as follows: denaturation at 95℃ for 10 min, followed by 50 cycles of amplification (95℃ for 10 sec, 60℃ for 30 sec) and cooling at 40℃ for 10 sec. All samples were tested through qPCR for both 5' and 3' regions of the ribosomal RNA gene for selection of single copy transgenic plants, the method used in this study was described by Wang et al [16].

### FST analysis

Genomic DNA extracted from each of the T_0_ transgenic plants were used to selected single copy transgenic plants and later used for inverse PCR adjacent sequencing of the inserted T-DNA. Using the FSTs (flanking sequence tags), the estimated position at which the T-DNA was introduced was determined after matching through blast program. IPCR (inverse PCR) is a method that depends on the results of digestion of the genome near the inserted T-DNA, using a specific restriction enzyme. Since the whole genome sequences of rice digested with *Bfa*I have around 500 bp gaps, an experiment was designed to check this restriction enzyme. To increase the recovery efficiency of FSTs, the FSTs reaction was performed in both left border (LB) and right border (RB) of the T-DNA. Primers suitable for amplification of the T-DNA from the FSTs analysis were made after testing the efficiency of each LB and RB associated primer based on the *Bfa*I adapter primer (S1 Table).

**Fig 2:**
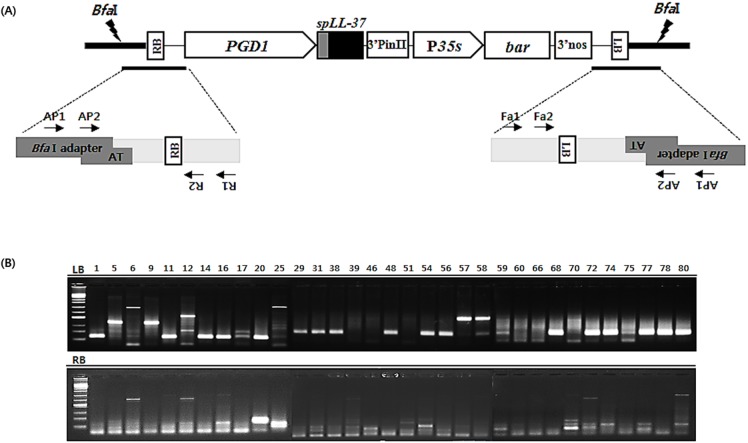
Analysis of flanking regions adjacent to the left or right border of T-DNA. (A) The diagram illustrates the PCR strategy used to obtain sequences flanking the T-DNA insertion sites. Restriction enzymes (shown as lightning) with recognition sites near the T-DNA border sequences (LB and RB) were used to digest DNA from the T-DNA insertion lines. Enzymes cut both within the T-DNA, and ligations were performed to circularize purified digestion products with *Bfa* I adapter. PCR1 using primers (S1 Table) specific to adapter and T-DNA (AP1+R1 for RB analysis or AP1 + Fa1 for LB analysis). PCR2 using nested primer pairs (AP2+R2 for RB analysis or AP2+Fa2 for LB analysis). (B) PCR2 products were separated by gel electrophoresis. The first lane of each row contains a molecular marker (size of bottom five bands: 200, 400, 600, 800 and 1,000 nt).

Ten μL of plant genomic DNA (up to 100 ng) was added to a fresh tube. The genomic DNA was then digested with *BfaI* by mixing 10 μL of each genomic DNA sample with 10 μL of a mixture containing H_2_O, NEB buffer 4 and *BfaI* in proportion. The samples were incubated at 37℃ for 3 h, after which the *BfaI* was inactivated at 65°C for 20 min. The *Bfa*I adapter was then ligated to the digested genomic DNA by mixing 20 μL of each digested DNA sample with 5 μL of a mixture containing H_2_O, NEB buffer 4, ATP, BSA, *Bfa*I adapter and T4 DNA ligase. The samples were then incubated overnight at room temperature (20 ~ 25℃), after which the T4 DNA ligase was inactivated by incubating the samples at 65℃ for 10 min. Next, 7 μL of adapter-ligated genomic DNA was transferred to a fresh tube/well, followed by addition of 43 μL of a mixture containing H_2_O, Taq DNA polymerase, buffer, dNTPs and primers. To amplify the regions flanking the RB of the T-DNA insert, AP1 and Fa1 or L1 primers were used. For amplification of the regions flanking the LB of the T-DNA insert, the AP1 and T-DNA primers were employed (S1 Table, Fig 2A).

### Subcellular localization of SP-LL-37

By fusing the C-terminus of SP-LL-37 and GFP gene, a pGWB5::SP-LL-37 vector was constructed. Tobacco leaf was transformed with pGWB5::SP-LL-37 using *Agrobacterium* mediated injection. Young apical leaves (5 ~ 6 leaves per plant) were infiltrated with recombinant *Agrobacterium* strains using a syringe (2 mL). Leaves were superficially wounded with a needle to improve infiltration. For constructs pGFPt and LL-37::GFP, a single zone on the opposite half of the leaves of *N*. *tobaccum* was infiltrated. The *Agrobacterium* strain containing pGFPt was used as a control. Three plants were agroinfiltrated at each time, and each experiment was repeated twice. The agroinfiltrated leaves were photographed at 36 ~ 48 h following infiltration.

The visual detection of GFP fluorescence was performed on agroinfiltrated leaves. The transformed leaves were photographed using a confocal Olympus FV500 equipped with a combined laser with an excitation of 488 nm and emission of 520 nm (Olympus, Tokyo, Japan).

### Inoculation of transgenic rice

*In planta* bacterial leaf blight assay was carried out using 20 plants each from T_2_ generation, as well as untransformed control plants, using ~1×10^7^ cells ml^-1^ of *X*. *oryzae* pv. oryzae following the ‘scissors clip-inoculation’ method [17]. A pair of scissors was dipped into the bacterial suspension and used to cut the leaf tips at 3/4 of an inch below the tip. The inoculated plants were then covered with polythene bags for 24 h and incubated at 30℃ with a 12 h light cycle. Inoculated plants were checked for water soaked areas within 48 to 72 h, and observations continued until 12 days.

To test the resistance of transgenic plants to rice blast, artificial inoculation and field evaluations were carried out. At four-leaf stage, seedlings were inoculated by spraying with a spore suspension of fungal strain in 0.02% Tween-20 with a concentration of 100 spores in 100× visual field. After inoculation, the seedlings were kept at 26 ~ 28°C with saturated humidity for 24 h in the dark, and then transferred to an artificial climate-tunnel at 26°C. Disease reactions were scored about 7 days after inoculation using the standard described by Pan et al., 1996 [18].

*M*. *oryzae* strain was selected because it causes partiality to Dongjinbyeo. Spores were obtained by treatment under a fluorescent lamp at 28°C for 48 hours, and then precipitated in sterile water after 7 days of incubation in oatmeal medium. The spore concentration of 10*10 per 100 x field was determined using a microscope and the spores were suspended with a concentration of 10^5^ CFU / ml. Spore suspension was made by addition of 0.2% Tween 20. Seedlings of at four-leaf stage were then inoculated by manual spraying.

### ELISA analysis for detection of LL-37 protein

Protein extraction and analysis of LL-37 expressing lines were performed as described by Alan *et al*., 2004 [19]. The total soluble protein was extracted from untransformed control plants (WT) and transgenic rice plants by homogenizing in extraction buffer and quantified by the Bradford method [20]. The levels of recombinant LL-37 protein were determined via direct ELISA assay. The ELISA plates were coated with 100 μg TSP in 0.05 M carbonate/bicarbonate (pH 9.6) buffer and then incubated with mouse IgG conjugated with horseradish peroxidase (secondary antibody), diluted 1:1,000 in blocking buffer, for 2 h at room temperature. To stop the reaction, 100 μL of tetramethylbenzidine substrate (Pierce, IL, USA) and H_2_O_2_ were added. After incubation, the reaction was measured at 405 nm with an ELISA reader.

## Results

### Generation and characterization of SP-LL-37 transgenic plants

Transgenic plants with SP-LL-37 were generated by using PPT as a selectable marker and transforming Dongjinbyeo embryogenic calli using *Agrobacterium*-mediated method. 472 (40%) transgenic plants from a total of 1,180 PPT resistant calli were regenerated (Fig 1C).

To verify the transgenic plants, PCR analysis with 472 plants was performed and our results showed positive amplification of SP-LL-37 and BAR genes. To select single copy plants, Taqman PCR was done using actin gene probe and NOS gene probe. 181 transgenic plants showed copy number values ranging from 0.42 to 0.65, thus displaying single copy (Fig 1D) (Table 1).

**Table 1:** Copy number frequency of pPZP::SP-LL-37 in transgenic plants.

Lines	TaqMan copy assay			Single copy (%)	Average of T-DNA inserts
	1 copy	2 copies	Multi copies		
472	181	61	230	38.3	2.1

### FSTs analysis using inverse PCR

**Fig 3:**
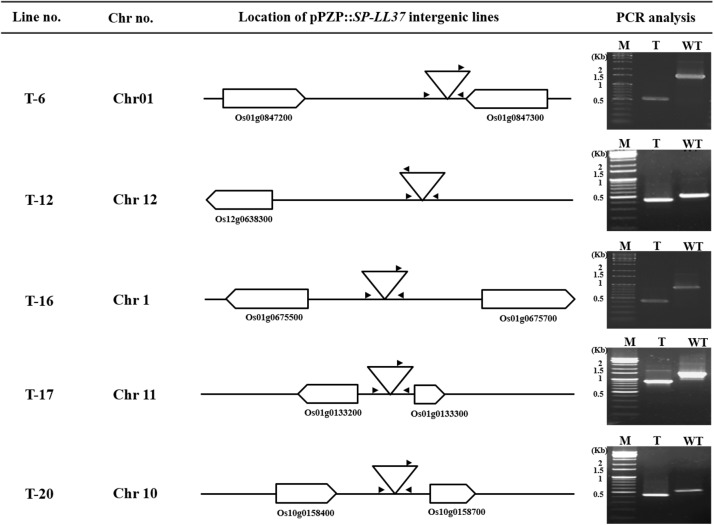
Identification of pPZP::SP-LL-37 insertion transgenic plants. The genomic structures of insertion alleles were determined by FST analysis in which boxes, bold lines, and triangles indicate exons, intron, and pPZP::SP-LL-37, respectively. The arrow and arrowhead indicate gene specific primer pairs from genomic DNA. Genomic DNA was isolated from the leaves of pPZP::SP-LL-37 regenerated plants for PCR analysis. WT: wild type, T: T_0_ plant with single T-DNA insert for pPZP::SP-LL-37 insertion line.

There were 121 T_0_ plants subjected to IPCR (inverse PCR) analysis using specific primers for *BfaI* adapter and T-DNA primers flanking the LB and RB sites (Fig 2A). The efficiency was low because only one restriction enzyme was used in the analysis. Therefore, upon detection of the band, either the LB or RB was used to secure the NCBI Blast primary site. If only the intergenic region was obtained, secondary analysis was performed. The FSTs secured from 121 lines displayed various size of amplicons ranging from 41 ~ 888 bp, with the highest proportion between 50 ~ 200 bp (Fig 2B). The position of the T-DNA, as determined from the FST analysis, was genic in 92 (77.7%), repeated sequences in 13 (10%) and intergenic in 14 cases (11.6%) (Table 2). Most of the obtained sequences contain a portion of the vector sequence, which did not match the known nucleotide sequence in NCBI, and when a short repeat sequence was measured, the sequence could not be read. Based on the nucleotide sequences of the FSTs, analysis through the NCBI BLASTn program allowed selection of 14 intergenic lines (Table 3). For accurate analysis of the 14 lines estimated to have intergenic location of the T-DNA, primers were made using the genome sequences obtained from BLASTn, targeting a specific site within 1kb. When performing PCR on the 14 lines using specific primers, the expected target size was obtained in five lines: T6, T12, T16, T17, and T20. The T-DNA fell between the Os01g0847200 and Os01g0847300 genes in Chr01 for T6, later than Os12g0638300 in Chr12 for T12, between Os01g0675500 and Os01g0675700 in Chr01 for T16, between Os01g0133200 and Os01g0133300 in Chr11 for T17, and between Os10g0158700 and Os10g0158400 in Chr10 for T20 (Fig 3). Five intergenic plants were used for expression analyses and agronomic performances.

**Table 2:** Frequencies of T-DNA insertions in the genic and intergenic regions.

Distribution of T-DNA inserts		T-DNA	
		No. of sequences	Ratio (%)
Genic	Exon region	32	26.4
	Intron region	23	19
	3' Downstream (500bp)	22	18.2
	5' Upstream (500bp)	17	14
Intergenic		14	11.6
Repeat (>1)		13	10.7
Total		121	100

**Table 3:** Flanking T-DNA analysis from pPZP::SP-LL-37 T_0_ plants.

Lines	Chr.	Query	Matching length	Chr. start	Chr. end	Type
5	chr 02	101	176	29,730,040	29,730,141	intragenic
11	chr 01	225	396	36,396,000	36,400,999	intergenic
12	chr 12	65	65	27,366,233	27,366,267	intergenic
17	chr 02	118	199	13,022,323	13,022,440	intergenic
20	chr 01	94	122	27,773,691	27,773,757	intergenic
25	chr 05	115	208	7,803,000	7,809,999	intragenic
31	chr 02	58	82	29,730,057	29,730,114	intragenic
48	chr 10	46	95	3,699,000	3,710,999	intergenic
54	chr 08	358	646	23,069,000	23,069,358	intragenic
57	chr 10	46	69	3,704,424	3,704,451	intergenic
58	chr 10	46	105	3,700,000	3,710,999	intergenic
59	chr 01	495	888	36,398,371	36,398,865	intergenic
70	chr 05	22	41	29,519,611	29,519,632	intergenic
72	chr 01	147	203	17,222,077	17,222,191	intergenic
74	chr 01	390	690	36,394,000	36,405,999	intergenic
77	chr 08	163	260	23,035,211	23,035,354	intragenic
80	chr 04	59	98	28,854,000	28,865,999	intergenic
81	chr 01	122	134	27,766,000	27,782,999	intergenic
82	chr 11	113	124	1,530,000	1,563,999	intergenic

### SP-LL-37 expression analysis of the T_1_ generation

**Fig 4:**
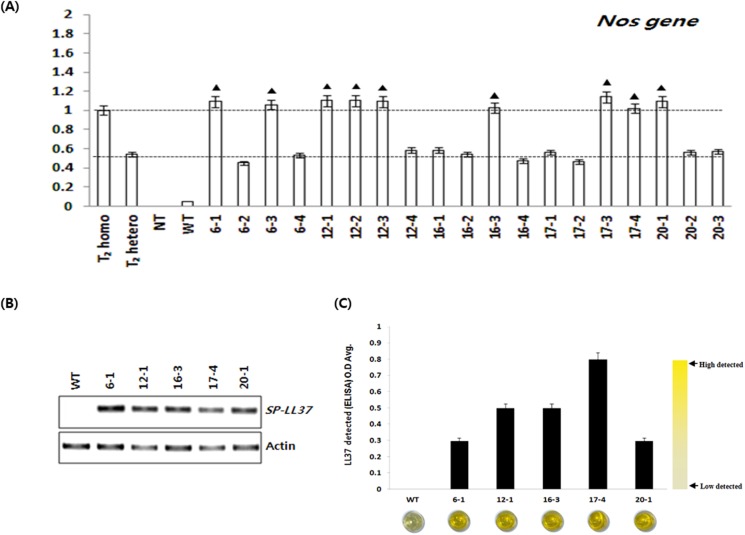
Generation and molecular analysis of the transgenic lines expressing pPZP::SP-LL-37. (A) TaqMan PCR analysis for copy number assays using TaqMan probe for selected homozygous T_1_ plants; T_2_ homo; T_2_-homozygous, T_2_ hetero; T_2_-heterozygous, NT; no template, WT; wild type, 6–1~20–3; 19 T_1_ plants. (B) SP-LL-37 gene expression in T_1_ homo transgenic lines using RT-PCR. Total RNA was isolated from each plant, and 0.5 *μg* of this RNA was amplified with SP-LL-37-specific primers. Rice actin gene was amplified as a loading control. (C) ELISA analysis of SP-LL-37 lines in bovine sperm conditioned media collected at T_1_-transgenic rice plants.

Nineteen plants in T1 generation derived from five T0 intergenic plants were used in the expression analysis. Rice plants were treated with 0.3% basta by spraying at three leaf stage. In one week, WT plants were yellowing and were completely dried later, while the transgenic plants maintained their green color. The plants treated with basta segregated into green (3):yellow (1) ratio. To select homo lines, a NOS gene probe was used in Taqman PCR for copy number analysis. The transgenic lines 6–1, 6–3, 12–1, 12–2, 12–3, 16–3, 17–3, 17–4, and 20–1 were all identified to have one copy number, and were therefore selected as homo lines (Fig 4A). Total RNA was extracted from the five homo lines selected for the analysis of LL-37 expression level (Fig 4B). Stable expression of LL-37 was confirmed in all five transgenic lines. Protein extracts from the leaves of five transgenic lines were then subjected to ELISA analysis. Fig 4C shows that LL-37 is detected in all transgenic plants and 17–4 line has the highest content among the transgenic plants.

### Cellular localization of SP-LL-37 protein

**Fig 5:**
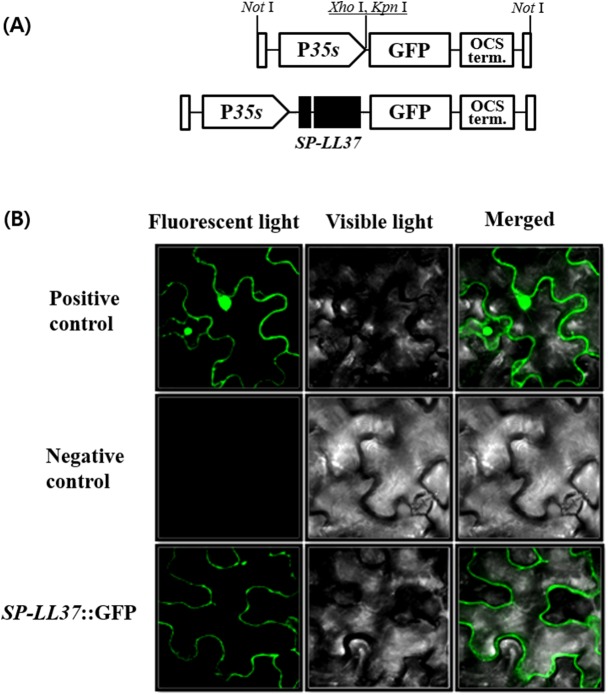
Subcellular localization of the SP-LL-37 protein in tobacco. GFP protein was attached to the end of C-terminal of LL-37 protein to see the localization in tobacco cells. **(A)** Cartoon image of constructs used in subcellular localization. **(B)** Fluorescence image of cell membrane (*N*. *benthamiana* epidermal cells) from *Agrobacterium*-mediated transient expression (using the p19 protein to enhance the expression level) and cytoplasm expressing the SP-LL-37 protein with positive and negative control by confocal microscopy.

When developing varieties producing antimicrobial peptides, stable expression must be considered. Since antimicrobial peptides are very short, they are susceptible to degradation by the proteases of the plant. LL-37 expression was maximized by using a signal peptide targeting it for secretion into the intercellular space, where it would have the least impact on the plants. To test whether the signal peptide indeed induced secretion of LL-37 from the nucleus to the intercellular space, subcellular localization by tobacco transient assay was performed. SP-LL-37::GFP fusion protein and a GFP only constructs (Fig 5A) were injected in tobacco. After four days of infection, subcellular pattern of accumulation was determined using confocal microscopy. The GFP signal of the SP-LL-37-GFP fusion protein was located in the intercellular space. Empty vector was expressed uniformly throughout the cell, and the GFP signal was not detected in the negative control (Fig 5B).

### Characterization of disease resistance and agronomic traits of transgenic SP-LL-37 lines in T_2_ generation

**Fig 6:**
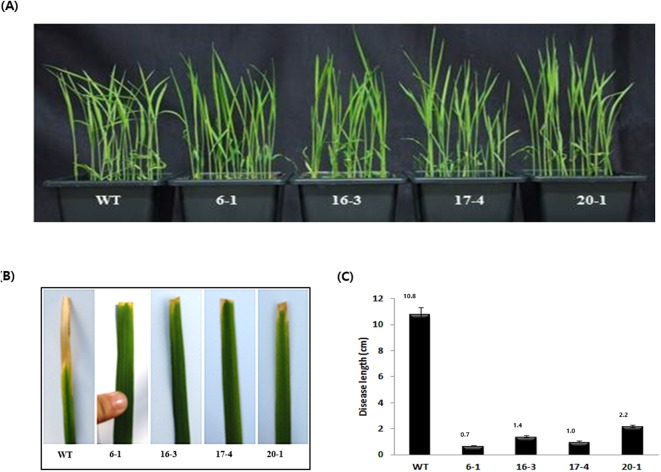
Identification and characterization of the T2 homozygous plants. **(A)** Phenotype of transgenic T_2_ generation in pot. **(B)** Whole plant infection assay of WT and T_2_ generation. Disease development in T_2_ generation inoculated with *X*. *oryzae* KACC10859 strain. **(C)** Disease scoring was conducted 10 days after inoculation. WT; Dongjinbyo rice, 1~5; T_2_ transgenic homo lines.

The SP-LL-37 lines displayed no phenotypic differences compared to their WT (Fig 6A). To analyze the effects of SP-LL-37 on resistance to pathogenic bacteria, transgenic lines (6–1, 16–3, 17–4, 20–1 and WT) were inoculated with *X*. *oryzae* KACC 10859 strain (K3a) (Fig 6B). The K3a inoculum was diluted to a concentration of 1 × 10^7^ cells/ml in LB liquid medium. Rice leaves were infected at three leaf stage of the plant. The SP-LL-37 transgenic plants showed less noticeable symptoms of infection than the WT. Three weeks later, the size of lesions on leaves of the WT had significantly increased to 10.8cm, while the transgenic lines showed very small lesions of 0.1 ~ 2.2 cm in size (Fig 6C).

**Fig 7:**
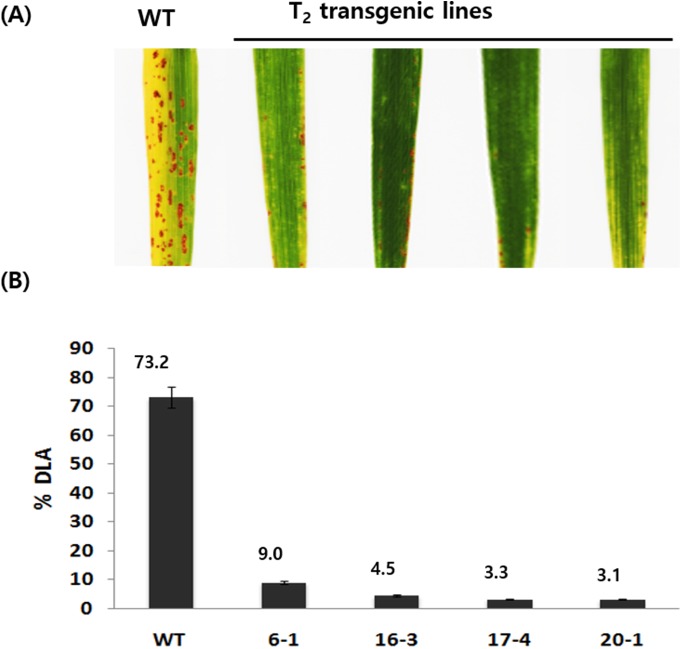
*In planta* bioassay of *M*. *oryzae* (10^5^ spores/ml) on transgenic lines. **(A)** Whole plant infection assay in WT and T_2_ transgenic homo lines. **(B)** Blast infected leaves of WT and T_2_ transgenic homo lines. Infected leaf area was measured in % DLA (Diseased leaf area) at 10 dpi. The data are presented as means ± SEM from three independent estimations.

In addition, to see if LL-37 has a broad spectrum of resistance, we inoculated the transgenic lines with blast fungus. Seven days after inoculation of rice blast strain, the percent frequency of diseased leaf area (DLA) was measured. Visually striking yellowish symptoms began to appear bordering the spindle in WT plants. From 14 days onward, the symptoms had spread throughout the leaf and were clear enough to distinguish the transgenic lines (Fig 7A). Approximately 73.2% of the total leaf area of WT plants was covered by lesions. However, in the four transgenic lines tested, substantial growth of the lesion was not observed, growing only from 3.1 to 9.0% (Fig 7B).

The transgenic SP-LL-37 homo lines obtained in this work were compared with WT in terms of agronomic traits (Table 4). No significant differences were found between transgenic lines and WT. Slight differences were observed between control plants and those with different transgenic lines. Most of the transgenic lines were a little shorter (range of plant height, 110.0±7.7 to 119.2±2.0 cm) than Dongjinbyeo plants (120.0±4.5 cm). Grain weight of transgenic line 73 (25.3±0.8 g/1000) was the same as control (25.9±0.1 g/1000). Line 93 also produced the same grain weight (24.6±0.7 g/1000) like wild type. The grain of other transgenic lines was little lighter (range, 20.8±0.6 to 24.3±1.2 g/1000) than that of wild type, although overall differences were in a very narrow range.

**Table 4:** Agronomic traits of pPZP:: SP-LL-37 T_1_ homo lines.

No.	Lines	Chlorophyll content (SPAD)	Days to flowering	Plant height	Culm length	Panicle length	Number of tillers	No. of spikelet/panicle	% filled grains	1000-grain wt.
1	Dongjinbyeo (WT)	40.8±0.1	103.7±1.0	120.0±4.5	86.7±2.6	21.0±1.5	11.3±3.6	161.3±5.5	93.0±0.7	25.9±0.1
2	17	41.0±3.1	104.7±1.0	110.0±7.7	79.4±4.7	19.3±1.0	10.3±5.2	160.0±4.5	90.1±0.1	22.5±1.0
3	20	44.1±0.7	102.3±0.5	111.7±6.8	83.3±2.6	18.5±1.2	16.7±4.9	146.0±5.0	88.0±0.6	23.5±1.2
4	42	41.1±1.8	105.0±0.9	117.7±6.1	74.0±4.7	21.7±1.4	10.0±5.0	149.6±4.2	82.4±0.5	22.4±1.1
5	73	45.8±1.3	104.0±0.9	113.3±9.3	78.3±2.9	20.3±0.5	9.0±1.8	156.8±3.5	87.6±0.2	25.3±0.8
6	84	44.1±1.8	103.3±0.5	113.7±9.8	78.7±4.9	23.4±2.8	11.0±4.1	157.0±5.8	89.6±0.4	22.1±0.4
7	93	40.2±2.3	114.7±1.4	119.7±1.9	79.3±4.9	20.3±1.4	9.0±0.9	148.7±5.8	84.4±0.5	24.6±0.7
8	96	41.3±0.6	112.6±1.2	116.5±1.5	79.3±2.9	17.3±1.5	10.6±4.4	160.2±4.6	88.8±0.4	21.6±0.8
9	102	42.1±1.9	106.6±1.1	114.9±1.4	80.0±2.3	18.6±1.6	10.6±5.9	148.2±3.5	92.1±0.5	23.6±0.9
10	107	42.7±0.8	105.6±1.0	115.8±1.3	75.5±3.0	14.3±0.9	9.7±6.6	150.8±2.0	91.6±0.8	22.4±3.6
11	111	43.5±0.4	111.0±2.0	117.7±6.5	82.3±3.3	18.5±0.9	9.7±4.4	158.5±5.0	90.6±0.8	21.6±1.3
12	119	42.5±0.3	103.5±0.5	116.4±4.0	83.3±4.0	20.9±0.7	9.3±4.7	160.2±6.5	92.4±0.6	20.8±0.6
13	121	40.4±0.5	102.5±0.4	118.8±3.0	85.2±6.0	22.8±0.6	9.4±3.8	154.6±3.0	92.4±0.2	23.1±0.2
14	132	41.5±0.2	103.4±0.8	119.2±2.0	84.6±3.0	21.8±0.7	8.6±2.6	151.5±4.0	84.2±0.1	22.0±1.5
15	137	44.5±0.8	102.5±0.7	118.2±1.0	81.6±4.6	20.9±0.9	9.2±3.4	156.9±3.0	90.3±0.3	24.3±1.2

## Discussion

Human cathelicidin antimicrobial protein LL-37 has attracted much attention because of its potential for broad spectrum antimicrobial activity, and the fact that it can be used as a substitute for antibiotics in the development of transgenic rice. We have already developed homozygous Chinese cabbage and tomato lines that stably expressed LL-37, which were found to be resistant to bacteria and fungi [13, 21]. Furthermore, LL-37 in transgenic rice controlled by a 35S promoter was resistant to bacteria (unpublished data). The purpose of this study was to develop a practical complex disease resistant transgenic rice using multifunctional LL-37. The expression of transgenes in plants is influenced by the transgene copy number, position effect, DNA methylation, and repeatability of the insertion site. It also cannot be ruled out that the insertion of transgene loci may cause stability problems to the plant itself, if disrupting certain parts of the genome [22]. Therefore, in contrast to other transgenic studies, we focused on a single copy and intergenic locations of transgenes, starting from T_0_ generation in order to ensure a stable expression.

Bacterial leaf blight inoculation experiments on four lines revealed significantly higher disease resistance than WT. The four transgenic lines showed lesions from 0.7 cm to 2.2 cm in size, which were smaller than the lesion length of 10.8 cm in WT. The transgenic plants did not show more signs of disease progression. In rice blast experiment, fusiform lesions were appeared as dark brown areas in over 73.2% of the control leaves, while substantial growth was not observed on the transgenic lines, which displayed lesions covering from 3.1 to 9.0% of the area. Therefore, the LL-37 gene product in the transgenic lines showed antibacterial activity against bacterial leaf blight and rice blast. In a different study, LL-37 has been reported to increase the expression of the PR-related protein in transgenic tomato, it increased the expression of defense-related gene response [21].

The expression of antimicrobial peptides in plants often fails due to the influence of proteases. For example, the Cecropin B peptide has a short persistence with the half-life of 25.5 h due to the effect of protease in transgenic rice [23]. To prevent peptide degradation by cellular enzymes, the vicilin gene was fused to the N-terminal as a signal peptide of LL-37 in transgenic rice. Results of the tobacco transient assay showed that SP-LL-37 was mainly located in the intercellular space of the plant (Fig 5B). This was the most effective strategy reported in the development of plants with antimicrobial peptides so far [24].

## Conclusions

Plant development for enhancing disease resistance is an important area to eliminate the damage caused by the widespread use of chemical pesticides. In this study, we showed how to minimize destruction to the rice genome by selecting single, intergenic lines among the SP-LL-37 transgenic lines. Furthermore, transgenic lines with SP-LL-37 showed enhanced resistance to bacterial leaf blight and blast fungus, achieved through stable expression and localization in the intercellular area using a signal peptide. The SP-LL-37 transgenic rice, line 17–4, should prove valuable to the use of AMPs to generate crops with wide spectrum of resistance to any disease or harmful environment.

## Supporting information

S1 Table List of oligonucleotides for flanking analysis. (DOCX)
