# Supplementary material for: SP-LL-37, human antimicrobial peptide, enhances disease resistance in transgenic rice
Source: PLoS One. 2017 Mar 10;12(3):e0172936. doi: 10.1371/journal.pone.0172936 (PMC5345758; doi:10.1371/journal.pone.0172936)
Supplement: S1 Table — (DOCX) [file pone.0172936.s001.docx]

**S1 Table . List of oligonucleotides for flanking analysis.**

| **Name** | **Description** | **Sequence (5'–3')** |
| --- | --- | --- |
| ADP2 | Used with ADP3 to generate the BfaI adapter | CTAATACGACTCACTATAGGGCTCGAGCGGCCGGGCAGGT |
| ADP3 | Used with ADP2 to generate the BfaI adapter | TAACCTGCCCAA |
| AP1 | Adapter primer used with R1 or F1 for first round of PCR | GGATCCTAATACGACTCACTATAGGGC |
| AP2 | Nested adapter primer used with R2 or F2 for second round of PCR | TATAGGGCTCGAGCGGC |
| R1 | T-DNA primer near RB used with AP1 for first round of PCR | TTA GCT TGA GCT TGG ATC AGA TTG TCG |
| R2 | Nested T-DNA primer near RB used with AP2 for second round of PCR | TCA GAT TGT CGT TTC CCG CC |
| Fa1 | T-DNA primer near LB used with AP1 for first round of PCR | CGG GAA TTC AAT TCG GCG TTA ATT CAG |
| Fa2 | Nested T-DNA primer near LB used with AP2 for second round of PCR | TTC AGT ACA TTA AAA ACG TCC GC |
